# Multicenter Cross-Sectional Study of Nontuberculous Mycobacterial Infections among Cystic Fibrosis Patients, Israel

**DOI:** 10.3201/eid1403.061405

**Published:** 2008-03

**Authors:** Isaac Levy, Galia Grisaru-Soen, Liat Lerner-Geva, Eitan Kerem, Hana Blau, Lea Bentur, Micha Aviram, Joseph Rivlin, Elie Picard, Anita Lavy, Yakov Yahav, Galia Rahav

**Affiliations:** * Sheba Medical Center, Tel Hashomer, Israel; † Gertner Institute for Epidemiology and Health Policy Research, Tel Hashomer, Israel; ‡ Hadassah Hebrew University Hospital, Jerusalem, Israel; § Schneider Children’s Medical Center of Israel, Petah Tikva, Israel; ¶ Rambam Medical Center, Haifa, Israel; # Soroka University Hospital, Beer Sheva, Israel; ** Carmel Medical Center, Haifa, Israel; †† Shaare Zedek Medical Center, Jerusalem, Israel; ‡‡ Mycobacterium Reference Laboratory of Israel, Tel Aviv, Israel; 1 These authors contributed equally to this work.; 2 Current affiliation: Dana Children’s Hospital, Tel Aviv Sourasky Medical Center, Tel Aviv, Israel.

**Keywords:** cystic fibrosis, nontuberculous mycobacteria, Mycobacterium simiae, Mycobacterium abscessus, research

## Abstract

This 2-year cross-sectional evaluation of nontuberculous mycobacterial (NTM) infections involved all Israeli medical centers that treat cystic fibrosis patients. The study comprised 186 patients whose sputum was analyzed for NTM. The prevalence of NTM isolates was 22.6%, and 6.5% and 10.8% of the patients fulfilled the 1997 and 2007 American Thoracic Society criteria for NTM lung disease, respectively. *Mycobacterium simiae* (40.5%), *M. abscessus* (31.0%), and *M. avium* complex (14.3%) were the most prevalent. Presence of *Aspergillus* spp. in sputum and the number of sputum specimens processed for mycobacteria were the most significant predictors for isolation of NTM (odds ratio [OR] = 5.14, 95% confidence interval [CI] 1.87–14.11 and OR = 1.47, 95% CI 1.17–1.85, respectively). The incidence of NTM pulmonary infections is increasing among cystic fibrosis patients, reflecting the increase in longevity of such patients as well as environmental exposure to various species of mycobacteria.

## Abstract 2

A multicenter cross-sectional study showed prevalence appears to be increasing.

The dramatic improvement in the survival of patients with cystic fibrosis (CF) has been complicated by the development of highly resistant strains of *Pseudomonas aeruginosa* and *Staphylococcus aureus*; the appearance of new virulent pathogens, such as *Burkholderia cepacia*; and the emergence of organisms of undetermined clinical importance, such as *Alcaligenes xylosoxidans*, *Stenotrophomonas maltophilia,* and the nontuberculous mycobacteria (NTM) (*1*–*4*). Since 1990, an increasing number of studies have reported the recovery of NTM from the respiratory tract of patients with CF at a prevalence of 2%–28% (*2*–*13*); higher prevelances have been reported in the United States (*14*) than in Europe (*4*,*11*). Whether such findings indicate infection or simply colonization of the airways by an environmental organism is not clear. The 1997 American Thoracic Society (ATS) guidelines for the diagnosis of NTM lung disease include compatible clinical and radiographic findings as well as bacteriologic findings of 3 positive cultures or, alternatively, 2 positive cultures and a positive smear for acid-fast bacilli (AFB) (*15*). The 2007 ATS microbiologic criteria, however, require the following: 1) 2 positive sputum cultures or 1 positive culture if it was obtained through bronchial wash, lavage, or lung biopsy; or 2) >1 sputum or bronchial washings that are culture positive for NTM if mycobacterial histopathologic features were evident (*16*). In a recent multicenter study of CF patients in the United States, Olivier et al. (*14*) reported an overall prevalence of NTM in sputum of 13%. Most isolates in their study were of the *Mycobacterium avium* complex (MAC), although a high prevalence of *M. abscessus* was also noted. Only 20% of the samples met the 1997 ATS microbiologic criteria for disease. It was suggested that patients with CF and multiple positive NTM cultures, characteristic high-resolution computerized tomographic (HRCT) findings, and progression of HRCT changes should be monitored closely and considered for antimycobacterial drug therapy (*17*).

In Israel, 468 patients with CF are currently treated in 7 medical centers. Although all 7 report that they screen for NTM pulmonary secretions on a regular basis and during most CF exacerbations, we wanted to investigate the various approaches they used to diagnose NTM pulmonary disease. We also wanted to determine the prevalence of NTM infection, the different species involved, and the associated risk factors for the development of NTM pulmonary infections in Israeli patients whose sputum was processed for NTM.

## Methods

### Definition and Ascertainment of Cases

This retrospective observational study was conducted at all Israeli medical centers that treat patients with CF. The medical records of all CF patients from July 2001 through July 2003 were screened. The number of patients ranged from 15 to 134 per center, with a total of 468 patients. Most patients routinely visited the centers in intervals of at least 3 months. The study population included CF patients >5 years of age who had not undergone lung transplantation and in whom sputum specimens were processed for mycobacteria.

We defined NTM infection as a patient having had at least 1 positive isolate over time. NTM disease was defined as the condition in which a patient had a positive NTM isolate and met ATS disease criteria. CF patients who were evaluated at least once without evidence of NTM constituted the control group. We analyzed the data according to the 1997 and 2007 ATS criteria. The study was approved by the respective institutional review boards.

### Data Collection and Study Design

The study design was cross-sectional. Demographic, clinical, and laboratory data for all eligible patients were collected from medical records, which included: age, gender, CF genotype, sweat chloride level, body mass index, forced expiratory volume in 1 s (FEV_1_; average during the study period), pancreatic function, presence of hemoptysis, sputum cultures during the study period, length of hospitalization (total time throughout the study period), antimicrobial agents administered (yes or no during the study period), and other treatment modalities.

### Laboratory Methods

Respiratory tract specimens were assessed in the local microbiology laboratories of each center. The methods were not standardized, but the laboratories operated according to recommendations by international expert groups (*18*). When NTM did grow, however, isolates were forwarded to the National Mycobacterium Reference Laboratory of Israel for further identification. Specimens were processed by standard methods and inoculated onto MB/BacT bottle (BacT/Alert System, bioMérieux, Marcy l’Etoile, France), a Lowenstein-Jensen slant, and a Middlebrook 7H11 selective agar plate (*19*–*21*). All inoculated media and broths were incubated at 36°C until growth was observed or up to 7 weeks. Direct smears and smears from colonies were stained with Ziehl-Neelsen stain. Species identification was performed by conventional biochemical methods and by determining antimicrobial drug susceptibility patterns using the resistance ratio method and Etest (Biodisk, Solna, Sweden) (*21*,*22*). MAC isolates were confirmed by using commercial RNA/DNA probes (Accuprobe, Gen-Probe, Inc., San Diego, CA, USA).

### Data Analysis

Prevalence of NTM was calculated as the ratio between the number of CF patients with at least 1 positive culture and the total study population. Univariate analysis for the comparison of cases and controls was performed by using Student *t* test for continuous variables and the χ^2^ test for categorical variables. Multivariate logistic regression analysis was performed to evaluate the effect of predicting variables for NTM-positive cases. Only variables that were significant in the univariate analyses (p<0.05) were included in the model (age, number of sputum specimens that were processed for mycobacteria, number of hospitalization days, number of days receiving antimicrobial agents, FEV_1_, presence of hemoptysis, growth of *Pseudomonas* or *Aspergillus* spp. in sputum, presence of allergic bronchopulmonary aspergillosis, and treatment with azithromycin or ibuprofen). Data were analyzed by using SAS Software, version 9.0 (Cary, NC, USA).

## Results

### Study Population

A total of 282 of the 468 eligible CF patients were excluded from the study: 203 did not have any sputum processed for mycobacteria, 59 were <5 years of age, 8 underwent lung transplantation, 2 had received immunosuppressive treatments, and follow-up was not available for the remaining 10 (Table 1). Sputum specimens were processed for mycobacteria for 265 (57%) patients. Four large centers (A, C, D, E, Table 1) evaluated 60%–80% of their CF patients for NTM, and 2 smaller centers (F, G, Table 1) evaluated 45% of their CF patients. One center (B, Table 1) did not evaluate its CF patients for NTM, and those patients were excluded from the study.

**Table 1:** Number of patients in all medical centers and their reasons for exclusion from study

Center	Total no. patients	Absence of mycobacterial culture (%)	Age <5 y	Other causes*	No. patients included
A	134	36 (27)	5	11	82
B	82	74 (90)	7	1	0
C	75	28 (37)	13	3	31
D	71	14 (20)	14	1	42
E	60	26 (43)	14	2	18
F	31	17 (55)	4	2	8
G	15	8 (54)	2	0	5
Total	468	203 (72)	59 (21%)	20 (7%)	186

*Other causes, lung transplantation, immunosuppressive therapy, dropped from follow-up.

For the patients whose sputum was processed for mycobacteria, the average number of sputum samples per patient during the study period was 3.1 ± 3.03. Patients whose sputum was evaluated for NTM (n = 265) were older and had markers of the severe form of disease compared to those whose sputum was not evaluated for mycobacteria (n = 203) (Table 2). A total of 186 study participants were eventually enrolled in the study; 42 had NTM infection or disease and 144 were controls. Twelve of the patients with NTM infection had NTM disease according to the 1997 ATS criteria; 20 patients had NTM disease according to the 2007 ATS criteria (p = 0.07).

**Table 2:** Comparison of patients included in the current study, patients tested for NTM, and patients not tested for NTM*

Parameter	Patients included in the current study (n = 186)	Patients tested for NTM (n = 265)	Patients not tested for NTM (n = 203)	p value
Age (mean ± SD)	20.51 ± 10.40	20.22 ± 10.53	13.99 ± 10.70	0.0001
Sex, F/M	74/112	105/160	86/117	0.54
Hemoptysis	22	23	4	0.002
FEV_1_, L/s (mean ± SD)	67.90 ± 22.09	65.18 ± 21.69	82.94 ± 16.20	0.0001
Pancreatic insufficiency	135	151	131	0.09
Hospitalization, d (mean ± SD)	21.77 ± 28.54	23.80 ± 32.44	8.44 ± 14.03	0.0001
Administration of antimicrobial agents, d (mean ± SD)	22.66 ± 47.51	19.98 ± 45.52	2.30 ± 10.28	0.0001
Azithromycin	104	114	51	0.001
Ibuprofen	7	15	6	0.16
Insulin	17	20	9	0.16
Systemic steroids	13	37	19	0.12
Inhaled steroids	96	106	105	0.01

*NTM, nontuberculous mycobacteria; SD, standard deviation; FEV_1_, forced expiratory volume in 1 s.

### Prevalence of NTM

The prevalence of NTM isolation among CF patients was 22.6% (42/186) (95% confidence interval [CI] 16.2–27.9). The prevalence of NTM varied by geographic location: no NTM were isolated from patients residing in northern Israel (center E, Table 1), whereas the prevalence was 24%–29% in hospitals located in central and southern Israel (centers A, C, D, F, Table 1), (Figure).

**Figure:**
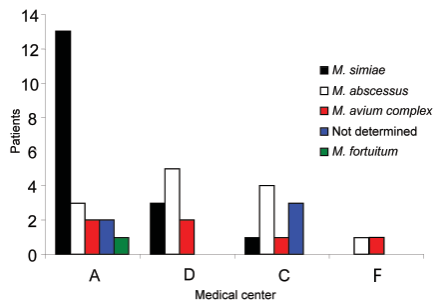
Different species of nontuberculous mycobacteria isolated from patients with cystic fibrosis (unique patient isolate) in 4 medical centers. *M*., *Mycobacterium*.

According to 1997 ATS criteria, 12 patients (6.5%) had NTM disease; 7 (58.3%) of these had AFB on smear. According to the 2007 ATS criteria, 20 patients (10.8%) had NTM disease, of whom 8 (40.0%) had AFB on smear. The proportion of patients with NTM disease in centers A, C, and D was 7.3%, 12.9%, and 4.8%, respectively, according to the 1997 ATS criteria, and 14.6%, 13.0% and 9.5%, respectively, according to the 2007 ATS criteria.

### NTM Species

The most common mycobacterial species were *M. simiae* (17 patients, 40.5%), *M. abscessus* (13 patients, 31.0%), and MAC (6 patients, 14.3%). *M. fortuitum* was isolated in 4 patients (2 patients had both *M. fortuitum* and *M. simiae,* and 1 patient had both *M. fortuitum* and *M. abscessus*). The species of NTM could not be determined for 5 other patients.

Species distribution differed according to geographic location (Figure). *M. simiae* was the most prevalent in center A, whereas *M. abscessus* was the most prevalent species in centers C and D. No mycobacteria were isolated in 2 centers. Seven of the 12 patients with NTM disease according to the 1997 ATS criteria were infected by *M. simiae* and 4 by *M. abscessus.* The species could not be determined for 1 patient. Nine of the 20 patients with NTM disease according to the 2007 ATS criteria were infected by *M. simiae*, 7 by *M. abscessus,* and 1 by MAC. The species could not be determined in 3 patients.

### Case-Control Study

Patients with NTM were significantly older (by 4.8 years) than culture-negative study participants, had more sputum specimens processed for mycobacteria, had more episodes of hemoptysis (23.8% vs. 8.3%), a lower FEV_1_ (14.5 L/s), a longer hospital stay (14.8 days), and more exposure to intravenous antimicrobial treatment (35.2 days) (Table 3). Patients with NTM were treated with azithromycin and ibuprofen more frequently. They had higher rates of *P. aeruginosa* (95.2% vs. 65.3% of controls) and *Aspergillus* spp. (66.7% vs. 21.5% of controls) in sputum samples. Both study and control groups were similar with respect to sex, sweat chloride level, pancreatic insufficiency, and requirement for insulin and steroids. Culture-negative and culture-positive groups had similar frequencies of mild and severe genotypes and also had the same rates of *S. aureus* and *S. maltophilia* growth. A multivariate analysis demonstrated that the presence of *Aspergillus* spp. in sputum and the number of sputum specimens processed for mycobacteria remained the only statistically significant predictors for developing NTM infection (odds ratio [OR] 5.14, 95% CI 1.87–14.11 and OR 1.47, 95% CI 1.17–1.85, respectively).

**Table 3:** Comparison of patients and controls in univariate and multivariate analyses*

Parameter	Patients (n = 42)	Controls (n = 144)	p value	Adjusted OR	95% CI
Age, y (mean ± SD)	24.2 ± 10.9	19.4 ± 10.0	0.014	1.03	0.99–1.09
Sex, F/M	18/24	56/88	0.64		
No. sputum specimens (mean ± SD)	5.7 ± 4.8	2.4 ± 1.7	<0.0001	1.47	1.17–1.85
Hemoptysis	10	12	0.006	1.08	0.29–4.05
FEV_1_, L/s (mean ± SD)	56.7 ± 19.6	71.2 ± 21.8	0.0001	0.97	0.94–0.99
Pancreatic insufficiency	35	100	0.07		
Sweat chloride, Meq/ L (mean ± SD)	73.1 ± 50.2	66.2 ± 48.3	0.43		
Hospitalization, d (mean ± SD)	33.2 ± 37.5	18.4 ± 24.5	0.019	0.99	0.97–1.01
Administration of antimicrobial drugs, d (mean ± SD)	49.9 ± 78.7	14.7 ± 29.4	0.007	0.99	0.98–1.00
Azithromycin treatment	30	74	0.02	1.00	0.99–1.00
Azithromycin treatment, d (mean ± SD)	367.6 ± 302.7	221.9 ± 288.4	0.007	1.00	0.99–1.00
Ibuprofen treatment	5	2	0.001	4.72	0.60–36.85
Insulin treatment	4	13	0.92		
Systemic steroids treatment	3	10	0.96		
Inhaled steroids treatment	26	70	0.13		
*Pseudomonas aeruginosa*	40	94	0.0005	0.76	0.32–1.79
*Staphylococcus aureus*	18	58	0.95		
*Aspergillus* spp.	28	31	<0.0001	5.14	1.87–14.11
Allergic bronchopulmonary aspergillosis	3	3	0.10		
*Haemophillus influenza*	3	24	0.13		
*Alcaligenes xylosoxidans*	0	5	0.22		
*Klebsiella pneumoniae*	3	4	0.17		
*Stenotrophomonas maltophilia*	2	3	0.34		

*OR, odds ratio; CI, confidence interval; SD, standard deviation; FEV_1_**,** forced expiratory volume in 1 s.

According to the 1997 ATS criteria, patients with NTM disease had more sputum specimens processed for mycobacteria, longer hospital stays, more courses of ibuprofen, higher isolation rate of *Aspergillus* spp., higher frequency of allergic bronchopulmonary aspergillosis, and more positive sputum smears than patients with NTM infection (Table 4). Analysis of the data according to the 2007 ATS criteria disclosed that patients with NTM disease had more sputum specimens processed for mycobacteria, used more inhaled steroids, and had more positive sputum smears. Patients with *M. abscessus* growth in sputum were younger than patients with the growth of other NTM (18.46 ± 6.42 vs. 26.79 ± 11.63 years, p<0.05), tended to have positive smears (53.8% vs. 13.8%, p<0.001), and had frequent growth of *S. maltophilia* in sputum (15.4% vs. 0, p<0.05). Patients with *M. simiae* growth in sputum had worse sweat test results than those with the growth of other NTM (92.18 ± 45.42 vs. 60.08 ± 49.88, p<0.05); they had more episodes of hemoptysis (52.9% vs. 12.0%, p<0.05), and they were treated more often with inhaled steroids (82.3% vs. 48.0%, p<0.05) and systemic steroids (29% vs. 0, p<0.01).

**Table 4:** Comparison of patients with NTM infection and NTM disease according to 1997 and 2007 ATS criteria*

Parameter	1997 criteria				2007 criteria		
	NTM disease (n = 12)	NTM infection (n = 30)	p value		NTM disease (n = 20)	NTM infection (n = 22)	p value
Age, y (mean ± SD)	19.8 ± 9.1	26.0 ± 11.2	0.17		24.2 ± 11.7	24.3 ± 10.4	0.97
Sex, F/M	7/5	11/19	0.20		9/11	9/13	0.79
No. sputum specimens (mean ± SD)	9.0 ± 5.1	4.4 ± 4.0	0.01		8.0 ± 5.2	3.6 ± 3.2	0.003
BMI, kg/m^2^ (mean ± SD)	18.9 ± 1.4	20.4 ± 4.0	0.19		19.7 ± 2.6	20.2 ± 4.1	0.63
Hemoptysis	4	6	0.36		6	4	0.37
FEV_1_, L/s (mean ± SD)	55.0 ± 23.0	57.4 ± 18.4	0.75		50.7 ± 20.3	62.1 ± 17.6	0.06
Pancreatic insufficiency	9	26	0.36		17	18	0.78
Sweat chloride (Meq/L) (mean ± SD)	66.5 ± 53.3	75.7 ± 49.6	0.61		81.9 ± 45.6	65.0 ± 53.7	0.28
Hospitalization, d (mean ± SD)	50.8 ± 52.5	26.2 ± 27.7	0.05		43.9 ± 44.1	23.6 ± 28.0	0.09
Administration of antimicrobial drug therapy, d (mean ± SD)	87.8 ± 110.9	34.7 ± 57.1	0.14		70.1 ± 90.6	31.5 ± 62.7	0.12
Azithromycin	10	20	0.55		15	15	0.63
Azithromycin treatment, d (mean ± SD)	379.5 ± 258.6	362.9 ± 322.7	0.87		360.7 ± 297.5	374.0 ± 314.2	0.89
Ibuprofen	4	1	0.01		4	1	0.12
Insulin	1	3	0.87		2	2	0.92
Systemic steroids	2	1	0.13		2	1	0.49
Inhaled steroids	10	16	0.07		16	10	0.02
AFB in sputum	7	4	0.01		8	3	0.05
*Pseudomonas aeruginosa*	12	28	0.36		20	20	0.17
*Staphylococcus aureus*	7	11	0.2		8	10	0.72
*Aspergillus* spp.	11	17	0.03		16	12	0.12
Allergic bronchopulmonary aspergillosis	3	0	0.004		3	0	0.06
*Hemophillus influenza*	2	1	0.14		2	1	0.52
*Klebsiella pneumoniae*	1	2	0.85		2	1	0.55
*Stenotrophomonas maltophilia*	2	0	0.02		2	0	0.14

*NTM, nontuberculous mycobacteria; ATS, American Thoracic Society; SD, standard deviation; BMI, body mass index; FEV_1_, forced expiratory volume in 1 s; AFB, acid-fast bacilli.

## Discussion

This multicenter study included 40% of the registered CF patients in Israel during a 2-year period and is the most representative study on NTM pulmonary infection among CF patients thus far. The only other comparable published report was a multicenter study from the United States by Olivier et al. (*14*), in which only 10% of the CF population was sampled. The overall prevalence of NTM in sputum in the current study was 22.6%, higher than the 13% reported from the United States (*14*). Twenty-six percent of the culture-positive study participants had positive smears, and one third to half of patients met the 1997 and 2007 ATS microbiologic criteria, respectively, for NTM disease. Half of the case-patients in our study had at least 2 positive cultures in contrast to 29% among the US CF population. The high prevalence in this study reflects the different mycobacterial ecology that exists in Israel. The findings also may be related to the 2-year cross-sectional design of this study compared to point prevalence studies. Furthermore, our study included patients for whom their physician had a reason to obtain a mycobacterial culture. This may have preselected a population with a higher likelihood of having mycobacteria. The study by Olivier et al. (*14*), which reported a lower prevalence, enrolled patients and prospectively obtained 3 sputum specimens over the course of 1 year.

There was some variability in the frequency of these bacteria between different centers. This finding may be due to differences in the quality of microbiology laboratories, differences in antimicrobial drug treatment, or endemic occurrence of bacteria in certain centers.

In the present study, *M. simiae* was the most common species isolated (40.5%), followed by *M. abscessus* (31.0%) and MAC (14.3%). *M. simiae* was also isolated among half of the patients with NTM disease. In the US study, MAC and *M. abscessus* were isolated in 72% and 16% of the cases, respectively. *M. simiae* is an infrequently found environmental organism that has rarely been associated with human disease. It has been reported as a cause of disseminated disease in AIDS patients (*23*–*25*) and the source of pulmonary disease in patients with underlying bronchiectatic lungs (*26*,*27*). To our knowledge, this is the first report on *M. simiae* pulmonary disease in CF patients. *M. simiae* was also associated with an outbreak due to a contaminated hospital water supply, which was distributed from an aquifer (*28*). Interestingly, the medical center with the highest prevalence of *M. simiae* did have an aquifer as the primary water source. Repeated water surveys, however, did not find contamination of facility water reservoirs as the source of the high prevalence of this species. *M. simiae* seems to have a limited geographic distribution; most clinical isolates have come from Arizona, New Mexico, Texas, Cuba, and Israel (*29*–*31*). In Israel, *M. simiae* is the most common NTM isolated from clinical specimens; the species usually colonizes damaged lungs (*30*,*31*). The distribution of *M. simiae* varied among treatment sites, with the highest incidence in central Israel (center A). In previous studies in Israel, ≈99% of *M. simiae* isolates were obtained from patients who resided on the coastal plain, mainly the Tel Aviv area (*30*).

As had been noted earlier by others (*14*), CF patients with NTM infection were older than those without NTM. In contrast to other studies, however, our CF patients with NTM infection had markers of severe disease, including lower FEV_1_, hemoptysis, higher frequency of *P. aeruginosa* and *Aspergillus* spp. growth in sputum, longer hospital stay, and higher exposure to intravenous antimicrobial agents, azithromycin, and ibuprofen. Mycobacterial colonization may be secondary to severe disease or that these mycobacteria cause the disease to be more severe. The association between NTM and markers of severe disease may be related to longer duration of disease or to the mycobacterial species involved. Severe disease could also promote altered mucociliary clearance, colonization, and infection with *M. simiae* and *M. abscessus*, while infection with MAC causes less progressive disease, mainly in older patients with mild disease (*14*).

In Olivier et al.’s study (*14*), *P. aeruginosa* was inversely associated with NTM, while the presence of *S. aureus* was positively associated with NTM. In our current study, patients with NTM tended to have less *S. aureus* and more *P. aeruginosa* in their lower airways. The strong association between infection with NTM and *Aspergillus* spp. probably reflects the severity of the disease. The presence of *Aspergillus* spp. or NTM may create favorable conditions for the colonization and infections of each other.

Azithromycin has a potential immunomodulatory effect in the treatment of CF, mainly for chronic *P. aeruginosa* respiratory tract infection (*32*). In our population, azithromycin was administered chiefly for its immunomodulatory properties and not to treat NTM pulmonary disease. Our study patients with NTM were treated more often with azithromycin (71.5% of case-patients vs. 51.4% of controls). Because macrolides are the treatment of choice for infections caused by MAC and *M. abscessus*, subtherapeutic doses of macrolides can induce selection of macrolide-resistant mycobacteria. The effect of long-term treatment with azithromycin on the antibimicrobial selection of NTM in CF patients remains undefined.

High doses of ibuprofen inhibit the inflammatory response to chronic infection, which contributes to lung destruction in patients with cystic fibrosis (*33*), and our patients with NTM were treated more frequently with ibuprofen. Furthermore, prostaglandin E inhibitors up-regulate the Th1 function with increasing levels of tumor necrosis factor, γ-interferon, and interleukin-2, which are necessary for the control of mycobacterial infections (*34*). The effect of prostaglandin inhibitors on mycobacterial infection has not been assessed in depth.

We did not find any correlation between the gene mutation profile and NTM infection. By contrast, others have demonstrated that 60.7% of patients with emerging bacteria were homozygous for the Delta F508 mutation in comparison to only 23.8% of the isolates from the control group (*4*).

Given the possibility that NTM may merely represent environmental contamination or simple colonization of the airways, we compared patients who were diagnosed as having NTM pulmonary disease with those with NTM infection, and found that, according to both 1997 and 2007 ATS criteria, those with NTM disease had more sputum specimens processed for mycobacteria and higher rates of positive smears. According to the 1997 criteria, patients with NTM disease had more severe pulmonary disease, and *P. aeruginosa* and *Aspergillus* spp. grew in their sputum more frequently.

The current high level of interest in NTM disease is the result of the recognition that NTM disease is encountered with increasing frequency in non-AIDS populations and in unrecognized settings with new manifestations. Furthermore, advances in mycobacteriology laboratories facilitated the publication of new diagnostic and therapeutic guidelines (*16*). The percentage of patients in our study who meet the current criteria is higher than those who met the previous criteria. It should be noted, however, that these guidelines apply to patients with lung disease due to MAC, *M. kansasii,* and *M. abscessus*, and it is not certain that these diagnostic criteria are universally applicable for all NTM respiratory pathogens.

This study has several limitations. First, retrospective studies can be limited by ascertainment bias, despite our best efforts to review all available paper and electronic records. Second, although 6 of the 7 centers report that they screen for NTM pulmonary secretions on a regular basis and during most exacerbations, only 45%–80% of patients in these centers were actually evaluated for the presence of NTM. Because testing for NTM was not routine in all centers, and since testing may have been performed preferentially on patients who showed clinical deterioration and in whom NTM-related disease was suspected, our data may not precisely reflect the overall prevalence of these bacteria in the population. Furthermore, an average of 6 sputum specimens were analyzed during the study period for each study patient while only 2.4 specimens were analyzed for each control. Nevertheless, this survey did provide some interesting insights about how often CF physicians look for NTM in sputum and gives an overview of the Israeli experience.

As the life expectancy of patients with CF increases and surveillance and microbiologic methods of detection improve, the prevalence of mycobacterial infection among the CF population appears to be increasing. The implication of this has not yet been conclusively established, and distinguishing between colonization and active disease remains difficult.
